# Infection Patterns and Predictors of 28-Day Mortality Among Cancer Patients in Najran, Saudi Arabia: A 10-Year Retrospective Cohort Analysis

**DOI:** 10.7759/cureus.107355

**Published:** 2026-04-19

**Authors:** Ahmed M Badheeb, Akram A Waddad, Ahmad Munajed, Ahmed Ibrahim, Saleh Y Algarea, Abdelraheem Elmubarak, Bassem D Alotaibi, Khaled A Alqfail, Ali Dhafer Al-Swedan, Omar Alkhanbashi, Ibrahim Hassan, Mohammed S Bazuqamah, Abdullah Abu Bakar, Esam Ben Yahya, Waleed Alselwi, Abdulrahman Mdsared

**Affiliations:** 1 Oncology, King Khalid Hospital, Najran, SAU; 2 Medicine and Surgery, King Khalid Hospital, Najran, SAU; 3 Medicine, King Khalid Hospital, Najran, SAU; 4 Pediatrics, Dongola Specialized Hospital, Dongola, SDN; 5 Internal Medicine, King Khalid Hospital, Najran, SAU; 6 Medicine, King Saud Medical City, Riyadh, SAU; 7 Medicine, Faculty of Medicine, Najran University, Najran, SAU; 8 Infectious Diseases, King Khalid Hospital, Najran, SAU; 9 Urology, King Khalid Hospital, Najran, SAU; 10 Cardiology, Prince Sultan Cardiac Center, Riyadh, SAU; 11 Laboratory Medicine, King Khalid Hospital, Najran, SAU; 12 Ophthalmology, King Khalid Hospital, Najran, SAU; 13 Medical Oncology, King Fahad Specialist Hospital, Dammam, SAU

**Keywords:** cancer, icu admission, infection, mortality, multidrug-resistant organisms, neutropenia, retrospective study, septic shock, survival analysis

## Abstract

Background and aim

Infections are a major cause of morbidity and mortality in cancer patients; however, regional data from southern Saudi Arabia remain scarce. The epidemiology of infectious complications can vary significantly due to differences in local pathogens, resistance patterns, and healthcare practices. Understanding these patterns is crucial for guiding empirical therapy and improving clinical outcomes. Therefore, this study aimed to characterize infection patterns and identify independent predictors of 28-day mortality among adult cancer patients in Najran, Saudi Arabia.

Methods

This retrospective cohort study at King Khalid Hospital Oncology Center (Najran, Saudi Arabia; January 2014 to December 2024) included 199 adult cancer patients (≥18 years) with 577 documented infection episodes. Primary outcomes were infection site distribution and microbiological profiles. Secondary outcomes included 28-day and overall mortality. The Andersen-Gill extension of the Cox model analyzed recurrent episodes, accounting for within-patient clustering. Multivariable Cox proportional hazards regression identified mortality predictors, reported as HRs with 95% CIs. ICU admission served as a proxy for clinical severity.

Results

Patients had a median age of 62 years; 133 (66.8%) had metastatic disease, and 100 (50.3%) were female. The most frequent malignancies were breast cancer in 35 (17.6%), colorectal cancer in 33 (16.6%), upper gastrointestinal cancers in 31 (15.6%), and hematologic malignancies (lymphoma/myeloma) in 30 (15.1%). Of 577 infection episodes, 351 (60.8%) were culture positive. Among positive cultures, Gram-negative organisms predominated, with *Klebsiella* (69; 12.0% of all episodes) and *Escherichia coli* (64; 11.1%) being the most common. Multidrug-resistant organisms accounted for 49 (8.5%) episodes. Among 199 patients, 179 (89.9%) survived, and 20 (10.1%) were non-survivors (28-day mortality). The 28-day mortality rate was 12.3% per episode. Independent predictors of mortality included ICU admission (HR 8.56, 95% CI 3.17-23.13, p < 0.001), neutropenia (HR 2.52, 95% CI 1.07-5.91, p = 0.034), noninvasive ventilation (HR 3.78, 95% CI 1.06-13.47, p = 0.041), and polymicrobial infection (HR 4.16, 95% CI 1.18-14.69, p = 0.027). Additionally, 440 (76.3%) episodes lacked a localized infection site, 106 (55%) patients had recurrent infections, and 137 (23.7%) required ICU admission. Culture-positive episodes trended toward higher ICU use (94 (26.8%) vs 45 (19.9%); p = 0.0765), but this was not statistically significant. Cause of death analysis showed underlying malignancy (51.0%) was more frequent than organ failure (38.5%; p = 0.068), with treatment-related complications (6.7%) and unknown causes (3.8%) contributing less.

Conclusions

In southern Saudi Arabia, Gram-negative pathogens and polymicrobial infections contribute significantly to mortality. The low multidrug resistance rate (8.5%) compared to other Saudi regions presents a critical opportunity for intervention. Local healthcare administration should urgently implement standardized diagnostic bundles and antimicrobial stewardship to maintain this favorable profile. Markers of acute deterioration (ICU admission, neutropenia, and noninvasive ventilation) strongly predict short-term mortality. These findings highlight the need for comprehensive clinical assessment and prospective studies with standardized definitions.

## Introduction

Infections remain a leading cause of treatment interruption and mortality in oncology, accounting for approximately 60% of deaths in this patient population [1]. The intersection of malignancy-related immunosuppression, cytotoxic chemotherapy, and frequent healthcare exposure creates a high-risk environment for infectious complications [2,3]. Importantly, these episodes are not restricted to patients with treatment-induced neutropenia; non-neutropenic cancer patients also face substantial infection risk. Recent evidence shows that non-neutropenic cancer patients presenting with fever require inpatient admission in up to 83% of cases, with the majority ultimately diagnosed with an infection [4]. In advanced solid tumors, infection-related mortality can exceed 47% [5], yet clinicians frequently struggle to balance the therapeutic benefits of antimicrobials against the complexities of drug-drug interactions and patient compliance.

The global epidemiology of oncology-related infections has shifted toward a predominance of Gram-negative bacteria and an increasing prevalence of multidrug-resistant (MDR) strains [1]. In the Gulf Cooperation Council (GCC) region, regional surveillance estimates the prevalence of extended-spectrum beta-lactamase (ESBL)-producing Enterobacterales at 21.6-29.3% in clinical samples [6]. Within Saudi Arabia, reports from high-acuity tertiary centers in Riyadh and Jeddah often document even higher resistance rates, with some oncology cohorts showing MDR proportions between 37.3% and 45% [7,8]. However, regional studies have documented variable patterns; for instance, while some centers report a high incidence of Gram-negative pathogens, others find Gram-positive bacteria as the most frequent isolates in specific subgroups. This variability underscores that a “one-size-fits-all” empirical therapy approach is unlikely to be optimal, particularly when mortality rates for sepsis in cancer patients are nearly ten times higher than in non-cancer populations [9].

In the Najran region, cancer epidemiology data exist [10-12], but oncology-specific infection patterns remain undocumented. The present study aims to evaluate infection characteristics and outcomes among cancer patients in this setting, with a focus on factors associated with short-term mortality. In addition, we perform stratified analyses based on culture status and neutropenia severity to explore potential differences in clinical severity. By integrating clinical, microbiological, and outcome data, this study seeks to provide a more nuanced understanding of infection-related risk in oncology patients while addressing key methodological challenges in this field.

## Materials and methods

Study design and setting

This retrospective cohort study was conducted at King Khalid Hospital Oncology Center in Najran, Saudi Arabia. We reviewed all adult patients (≥18 years) with a confirmed malignancy and at least one documented infection episode between January 2014 and December 2024. A clinical infection was defined as the presence of fever (temperature >38.3°C) or hypothermia (<36.0°C), along with clinical signs of infection (e.g., tachycardia, tachypnea, and leukocytosis or leukopenia) and either a positive culture or radiographic evidence of infection, after excluding noninfectious causes such as tumor fever or drug reactions [13]. Patients with incomplete documentation regarding infection onset or primary outcomes were excluded. The final cohort included 199 patients contributing 577 episodes.

Data collection and variables

Data were extracted from electronic medical records and the Oasis database. Demographic variables included age, sex, and patient source (internal vs. referred from outside). Cancer characteristics included primary site (categorized into 11 groups), stage (metastatic vs. nonmetastatic), and anticancer therapy type (single vs. combination). Combination anticancer therapy primarily referred to dual-agent chemotherapy (e.g., platinum-based doublets) or chemoradiotherapy.

Comorbidity burden was assessed by calculating an unweighted sum (range 0-12) of the following predefined conditions: diabetes mellitus, hypertension, chronic kidney disease (renal failure), ischemic heart disease, chronic obstructive pulmonary disease or asthma, heart failure, thyroid disorders, obesity, dyslipidemia, chronic liver disease, and rheumatic diseases. Infection details captured the date of onset, the site of infection coded according to adapted CDC/National Healthcare Safety Network surveillance definitions, and hospital-acquired status [14]. Hospital-acquired infection was defined as infection onset >48 hours after hospital admission. “Unknown site” was defined as a clinical infection in which no focal localization was identified via physical examination or imaging, regardless of culture status. For all episodes with an unknown/unspecified site, we recorded whether blood cultures were obtained and whether imaging (CT, MRI, or ultrasound) was performed.

Neutropenia was defined as an absolute neutrophil count (ANC) below 1500/μL and was further categorized by severity as mild (1000-1499/μL), moderate (500-999/μL), and severe (<500/μL) [15]. Severe neutropenia (<500/μL) was examined in sensitivity analyses due to its recognized clinical importance. Microbiology data included culture results and specific isolates. Polymicrobial infection was defined as the isolation of two or more organisms, while resistant infection was defined by the presence of methicillin-resistant *Staphylococcus aureus*, vancomycin-resistant enterococci (VRE), or MDR organisms (MDRO).

ESBL testing was performed on a subset of *Escherichia coli* and *Klebsiella *isolates based on clinical discretion (e.g., when resistance was suspected or for epidemiological surveillance). Data on antifungal prophylaxis and granulocyte colony-stimulating factor (G-CSF) usage were not available. Additional missing variables include prior antibiotic exposure, timing of recent chemotherapy, and baseline performance status. Regarding data quality, missing data for all primary variables were less than 5%, so multiple imputation was not performed.

Outcomes

The primary outcome was characterization of infection patterns, including site distribution and microbiological profiles. The secondary outcome was 28-day all-cause mortality.

Statistical analysis

Patient characteristics were summarized as frequencies and percentages for categorical variables, and medians with IQRs or means with SDs for continuous variables, as appropriate. Between-group comparisons (survivors vs. non-survivors) were performed using the chi-square test, Fisher’s exact test, or the Mann-Whitney U test, as appropriate.

Patient-level analyses were restricted to the first infection episode. Univariate Cox proportional hazards regression was used to estimate HRs and 95% CIs. Variables with p < 0.20 in univariate analysis were candidates for multivariable models. Collinearity was assessed using Spearman correlation (r > 0.7 excluded). Multivariable models used backward selection and robust standard errors for patient-level clustering. Model performance was evaluated using the concordance index (C-index).

For recurrent event analysis, we employed the Andersen-Gill extension of the Cox model to incorporate all 577 episodes while accounting for within-patient correlation. Sensitivity analyses excluded episodes with unknown infection sites or culture-negative episodes; results remained stable. Kaplan-Meier survival curves with log-rank tests were used to compare 28-day mortality across subgroups (metastatic status, neutropenia, terminal illness, and known infection site) using first-episode-per-patient data. Furthermore, exploratory univariate comparisons of 28-day mortality and ICU admission by antibiotic regimen (piperacillin/tazobactam, carbapenem, and number of antibiotics) were performed using the chi-square test. All analyses were performed using R version 4.4.0 (R Core Team, Vienna, Austria) with the survival package (version 3.5-7) and IBM SPSS Statistics for Windows, version 27.0 (released 2020; IBM Corp., Armonk, NY, USA).

Ethical approval

The study received approval from the Institutional Review Board of King Khalid Hospital (approval number H 11 N 182; approved March 15, 2023). Informed consent was waived due to the retrospective use of anonymized data.

## Results

Baseline and infection characteristics

**Figure 1:**
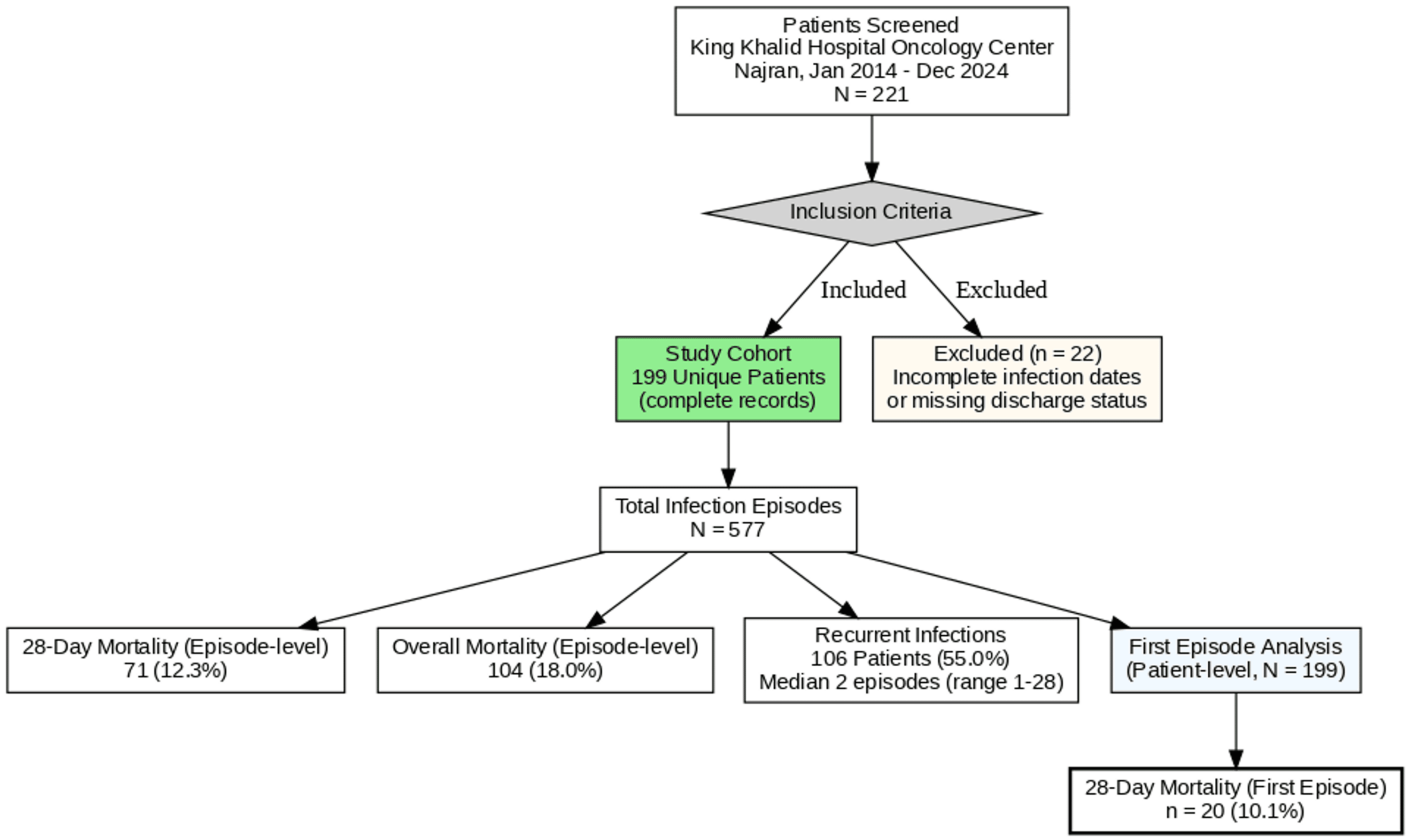
Study selection flowchart Of 221 unique cancer patients screened, 22 (10.0%) were excluded due to incomplete infection documentation, yielding a final cohort of 199 patients contributing 577 infection episodes. Patient-level analyses used the first infection episode per patient (N = 199); episode-level analyses included all 577 episodes. Recurrent infections occurred in 106 patients (55%), with a median of two episodes per patient (range 1-28). Figure created using Python (Matplotlib).

A total of 577 infection episodes were documented among 199 unique cancer patients (Figure 1). Median follow-up was 6.7 days (IQR 3-15) for first episodes and 26.9 days (IQR 8-64) for all episodes. At the patient level, 179 patients (89.9%) survived to 28 days, while 20 (10.1%) died.

The median age at first infection was 62 years (IQR 48-75), and 100 (50.3%) were female. The most frequent malignancies were breast cancer in 35 (17.6%) patients, colorectal cancer in 33 (16.6%) patients, and upper gastrointestinal cancers in 31 (15.6%) patients. Hematologic malignancies (lymphoma or myeloma) were present in 30 (15.1%) patients and metastatic disease in 133 (66.8%) patients. The most common comorbidities were diabetes in 64 (32.2%) patients and hypertension in 52 (26.1%) patients. Renal failure was present in 38 patients (19.1%), ischemic heart disease in 22 (11.1%), thyroid disease in 16 (8.0%), COPD or asthma in 12 (6.0%), and heart failure in nine (4.5%). Less common comorbidities included obesity (7, 3.5%), liver disease (7, 3.5%), dyslipidemia (3, 1.5%), rheumatic disease (3, 1.5%), and other conditions (5, 2.5%). The median modified comorbidity score was 2 (IQR 1-3).

There were no statistically significant differences in baseline characteristics between 28-day survivors and non-survivors, although non-survivors tended to have higher proportions of breast cancer (6, 30.0%) or gynecologic malignancies (5, 25.0%) (p = 0.068) (Table 1).

**Table 1:** Baseline demographics and comorbidities of cancer patients by 28-day survival status (patient level, first episode, N = 199) Data are presented as n (%) unless otherwise indicated. Patient-level analysis using the first episode per patient. Statistical tests: ^†^ Fisher’s exact test; ^‡^ Mann-Whitney U test KKHN, King Khalid Hospital Najran

Variable	Total (N = 199)	Survivors (n = 179)	Non-survivors (n = 20)	p-Value
Age, median (IQR)	62 (48-75)	63 (49-76)	55 (46-65)	0.26^‡^
Age group				0.401^†^
18-47 years	47 (23.6)	41 (22.9)	6 (30.0)	
48-63 years	57 (28.6)	49 (27.4)	8 (40.0)	
≥64 years	93 (46.7)	87 (48.6)	6 (30.0)	
Sex				0.632^†^
Female	100 (50.3)	88 (49.2)	12 (60.0)	
Male	98 (49.2)	90 (50.3)	8 (40.0)	
Patient source				1.0^†^
KKHN internal	144 (72.4)	130 (72.6)	14 (70.0)	
Referred from outside	55 (27.6)	49 (27.4)	6 (30.0)	
Cancer group				0.068^†^
Breast	35 (17.6)	29 (16.2)	6 (30.0)	
Colorectal	33 (16.6)	30 (16.8)	3 (15.0)	
Upper GI	31 (15.6)	30 (16.8)	1 (5.0)	
Lymphoma/myeloma	30 (15.1)	29 (16.2)	1 (5.0)	
Gynecologic	20 (10.1)	15 (8.4)	5 (25.0)	
Genitourinary	17 (8.5)	16 (8.9)	1 (5.0)	
Other	33 (16.6)	30 (16.8)	3 (15.0)	
Cancer stage				0.189^†^
Nonmetastatic	65 (32.7)	62 (34.6)	3 (15.0)	
Metastatic	133 (66.8)	116 (64.8)	17 (85.0)	
Diabetes mellitus	64 (32.2)	58 (32.4)	6 (30.0)	1.0^†^
Hypertension	52 (26.1)	48 (26.8)	4 (20.0)	0.602^†^
Modified comorbidity score, median (IQR)	2 (1-3)	2 (1-3)	2 (2-3)	0.74^‡^

Infection characteristics

Infection characteristics at the episode level are shown in Table 2. A clinically localized infection site was absent in 440 episodes (76.3%). Among the 440 episodes with an unknown/unspecified site, 254 (57.7%) had a positive blood culture, and imaging (CT, MRI, or ultrasound) was performed in 312 (70.9%); among those imaged, a focal source was identified in 98 (31.4%). Among localized cases, urosepsis (63, 10.9%) and pneumonia (33, 5.7%) were the most frequent. Hospital-acquired infections accounted for 274 episodes (47.5%). Neutropenia (ANC <1500/μL) was recorded in 66 episodes (11.4%).

**Table 2:** Infection characteristics and microbiological profiles of documented infection episodes by 28-day survival status (episode level, N = 577) Data are presented as n (%). Episode-level analysis accounting for clustering by patient. Statistical tests: ^*^ chi-square test; ^**^ Fisher’s exact test ^***^ “Unknown/unspecified” includes episodes with no clear anatomical focus. Among these 440 episodes, 254 (57.7%) had a positive blood culture; imaging was performed in 312 (70.9%), and a focal source was identified in 98/312 (31.4%). ^†^ Pneumonia includes “pneumonia” (n = 31) and “pneumonia, community-acquired” (n = 2). ^‡ ^Device-related includes “device-related catheter-associated urinary tract infection” (n = 7) and “device-related central line-associated bloodstream infection” (n = 5). ^§^ Intra-abdominal includes “biliary sepsis” (n = 1), “bed sore” (n = 2), and other intra-abdominal sources. ^¶^ Hospital-acquired infection defined as onset >48 hours after admission. ^#^ Percentages exceed 100% due to polymicrobial infections. Only selected organisms are shown. MDRO, multidrug-resistant organism; MRSA, methicillin-resistant *Staphylococcus aureus*; VRE, vancomycin-resistant *Enterococcus*

Variable	Total (N = 577)	Survivors (n = 506)	Non-survivors (n = 71)	p-Value
Infection site (grouped)				0.008^**^
Unknown/unspecified^***^	440 (76.3)	391 (77.3)	49 (69.0)	
Urosepsis	63 (10.9)	57 (11.3)	6 (8.5)	
Pneumonia^†^	33 (5.7)	31 (6.1)	2 (2.8)	
Skin/soft tissue	11 (1.9)	11 (2.2)	0 (0.0)	
Device-related^‡^	12 (2.1)	12 (2.4)	0 (0.0)	
Intra-abdominal^§^	11 (1.9)	8 (1.6)	3 (4.2)	
Surgical site	7 (1.2)	6 (1.2)	1 (1.4)	
Hospital-acquired infection^¶^	274 (47.5)	228 (45.1)	46 (64.8)	0.003^*^
Neutropenia	66 (11.4)	54 (10.7)	12 (16.9)	0.179^*^
Positive culture	351 (60.8)	304 (60.1)	47 (66.2)	0.39^*^
Organisms (selected)^#^				
Klebsiella	69 (12.0)	60 (11.9)	9 (12.7)	0.997^*^
Escherichia coli	64 (11.1)	53 (10.5)	11 (15.5)	0.289^*^
Candida	48 (8.3)	39 (7.7)	9 (12.7)	0.234^*^
Pseudomonas	35 (6.1)	29 (5.7)	6 (8.5)	0.526^*^
MDRO	24 (4.2)	19 (3.8)	5 (7.0)	0.326^*^
Acinetobacter	17 (2.9)	14 (2.8)	3 (4.2)	0.453^**^
VRE	24 (4.2)	20 (4.0)	4 (5.6)	0.52^*^
MRSA	1 (0.2)	1 (0.2)	0 (0.0)	1.0^**^
Any resistant organism (MDRO/VRE/MRSA)	49 (8.5)	40 (7.9)	9 (12.7)	0.18^*^
Polymicrobial infection	42 (7.3)	33 (6.5)	9 (12.7)	0.104^*^
Metastatic stage	391 (67.8)	327 (64.6)	64 (90.1)	<0.001^*^

**Figure 2:**
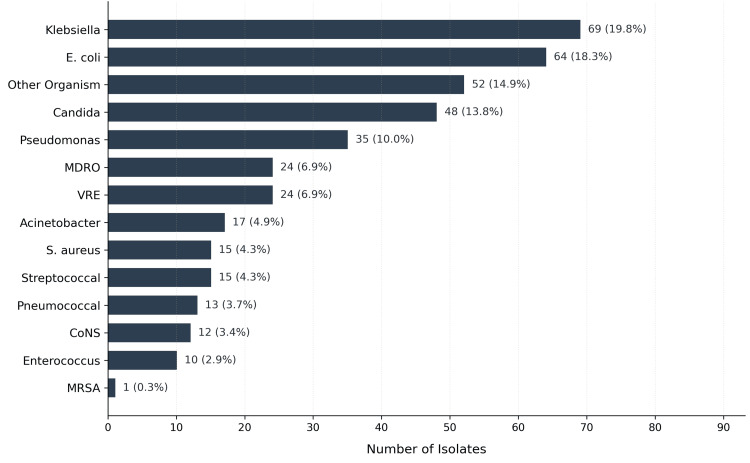
Distribution of isolated organisms among culture-positive episodes (N = 351) Bar chart showing the frequency of each organism type isolated from culture-positive infection episodes. A total of 351 episodes yielded positive cultures; some episodes yielded more than one organism; therefore, the total number of isolates exceeds the number of episodes. “Other organisms” (n = 52) includes *Proteus mirabilis *(8; 2.3%), diphtheroids (6; 1.7%), *Staphylococcus hominis *(4; 1.1%), *Enterobacter cloacae *(4; 1.1%), contaminant/mixed growth (6; 1.7%), *Staphylococcus haemolyticus (*3; 0.9%), *Staphylococcus capitis (*2; 0.9%), *Serratia marcescens *(2; 0.6%), unspecified other (2; 0.6%), and one each (0.3%): *Salmonella*, *Shigella*, *Vibrio cholerae*, *Staphylococcus epidermidis*, *Staphylococcus kloosii*, *Aeromonas caviae*, *Burkholderia cepacia*, *Clostridioides difficile*, *Chromobacterium violaceum*, COVID-19, herpes zoster, and Gram-positive cocci NOS. CoNS, coagulase-negative staphylococci; MDRO, multidrug-resistant organism; MRSA, methicillin-resistant *Staphylococcus aureus*; NOS, not otherwise specified; VRE, vancomycin-resistant *Enterococcus*

Among the 577 episodes, 351 (60.8%) were culture-positive, while 226 (39.2%) were culture-negative. The distribution of isolated bacterial species is shown in Figure 2, with *Klebsiella *spp. (69, 12.0%), *E. coli *(64, 11.1%), and *Candida *spp. (48, 8.3%) being the most common. MDROs were identified in 49 episodes (8.5%). ESBL testing was performed on 42* E. coli *and *Klebsiella *isolates (42, 31.6% of all such isolates), of which 12 (28.6%) were ESBL-producing, a finding subject to selection bias. Non-survivors had significantly higher rates of hospital-acquired infection (46, 64.8% vs. 228, 45.1%; p = 0.003) and metastatic disease (64, 90.1% vs. 327, 64.6%; p < 0.001). Polymicrobial infections trended higher among non-survivors (9, 12.7% vs. 33, 6.5%; p = 0.104).

Clinical outcomes

Clinical outcomes are summarized in Table 3. ICU admission occurred in 137 episodes (23.7%) and was significantly more frequent among non-survivors (43, 60.6% vs. 94, 18.6%; p < 0.001). ICU admission is best interpreted as a marker of illness severity rather than a baseline risk factor. Organ failures were notably prevalent in non-survivors, including requirements for invasive ventilation (33, 46.5% vs. 47, 9.3%), noninvasive ventilation (11, 15.5% vs. 15, 3.0%), and vasopressors (19, 26.8% vs. 6, 1.2%) (all p < 0.001). Do-not-resuscitate (DNR) orders were present in 48 non-survivors (67.6%) compared to 30 survivors (5.9%) (p < 0.001).

**Table 3:** Clinical interventions and severity of illness by 28-day survival status (episode level, N = 577) Data are presented as n (%) unless otherwise indicated. Episode-level analysis accounting for clustering by patient. Statistical tests: ^*^ chi‑square test; ^†^ Fisher’s exact test; ^‡^ Mann-Whitney U test ^§^ Length of stay data available for 556 episodes (96.4%). DNR, do-not-resuscitate; LOS, length of stay

Variable	Total (N = 577)	Survivors (n = 506)	Non-survivors (n = 71)	p-Value
ICU admission	137 (23.7)	94 (18.6)	43 (60.6)	<0.001^*^
Invasive ventilation	80 (13.9)	47 (9.3)	33 (46.5)	<0.001^*^
Noninvasive ventilation	26 (4.5)	15 (3.0)	11 (15.5)	<0.001^*^
Any ventilation	99 (17.2)	62 (12.3)	37 (52.1)	<0.001^*^
Inotropes	45 (7.8)	25 (4.9)	20 (28.2)	<0.001^*^
Vasopressors	25 (4.3)	6 (1.2)	19 (26.8)	<0.001^†^
Organ failure count, median (IQR)	0 (0-0)	0 (0-0)	1 (0-2)	<0.001^‡^
DNR order	78 (13.5)	30 (5.9)	48 (67.6)	<0.001^*^
LOS, median days (IQR)^§^	8 (5-8)	8 (4-8)	8 (8-8)	0.12^‡^

Cause of death attribution

Among the 104 total deaths, documented causes were cancer progression (53; 51.0%), organ failure (40; 38.5%), treatment complications, (7; 6.7%), and unknown (4; 3.8%). Infection-attributable mortality could not be reliably separated from malignancy-related organ failure.

Predictors of mortality

Univariate analysis (Table 4) identified ICU admission (HR 9.52), organ failure count (HR 2.84 per unit), polymicrobial infection (HR 5.10), and ventilation requirements as the strongest predictors (all p < 0.001). Neutropenia (HR 2.49, p = 0.062) and metastatic stage (HR 3.04, p = 0.076) showed trends.

**Table 4:** Univariate Cox proportional hazards analysis of predictors for 28-day and overall mortality (patient level, first episode, N = 199) Patient-level analysis using the first episode per patient. Statistical test: Cox proportional hazards regression (Wald test for p-values) NE, not estimable (zero events in one comparison group)

Variable	28-day mortality			Overall mortality		
	HR	95% CI	p-Value	HR	95% CI	p-Value
Age (years)	0.99	0.96-1.01	0.337	0.99	0.97-1.01	0.526
Male sex	0.67	0.27-1.64	0.378	0.81	0.38-1.74	0.596
ICU admission	9.52	3.79-23.91	<0.001	8.35	3.85-18.07	<0.001
Diabetes mellitus	0.92	0.35-2.39	0.862	1.09	0.49-2.44	0.826
Hypertension	0.7	0.23-2.10	0.528	0.82	0.33-2.03	0.667
Neutropenia	2.49	0.96-6.47	0.062	2.44	1.07-5.58	0.034
Hospital-acquired infection	1.06	0.43-2.59	0.9	1.06	0.49-2.28	0.885
Positive culture	1.63	0.65-4.10	0.295	1.5	0.69-3.27	0.31
Lactic acid >2 mmol/L	3.03	0.89-10.34	0.077	2.98	1.03-8.62	0.044
Invasive ventilation	6.97	2.68-18.16	<0.001	NE	-	-
Noninvasive ventilation	4.85	1.12-20.96	0.034	5.48	1.65-18.24	0.006
Any ventilation	7.65	3.12-18.75	<0.001	NE	-	-
Inotropes	NE	-	-	13.46	6.06-29.89	<0.001
Metastatic stage	3.04	0.89-10.38	0.076	3.44	1.19-10.00	0.023
Polymicrobial infection	5.1	1.70-15.26	0.004	3.9	1.34-11.30	0.012
Organ failure count (per unit)	2.84	2.00-4.05	<0.001	3.09	2.28-4.20	<0.001

In the multivariable Andersen-Gill model (Table 5), factors independently associated with 28-day mortality were ICU admission (as a severity marker; adjusted HR 8.56, 95% CI 3.17-23.13, p < 0.001), neutropenia (adjusted HR 2.52, 95% CI 1.07-5.91, p = 0.034), noninvasive ventilation (as a marker of respiratory failure; adjusted HR 3.78, 95% CI 1.06-13.47, p = 0.041), and polymicrobial infection (adjusted HR 4.16, 95% CI 1.18-14.69, p = 0.027). Metastatic status was not significant (adjusted HR 1.90, p = 0.318). The model showed good discrimination (C-index = 0.82).

**Table 5:** Multivariable Andersen-Gill model for recurrent events: independent predictors of 28-day mortality (episode level, N = 510 complete episodes) Andersen-Gill model accounting for recurrent episodes with robust standard errors for within-patient clustering. Variables with univariate p < 0.2 were included. Collinear variables (r > 0.7) were removed. Harrell’s C-index = 0.82. Statistical test: Wald test from Cox regression

Variable	Adjusted HR	95% CI	p-Value
ICU admission	8.56	3.17-23.13	<0.001
Neutropenia	2.52	1.07-5.91	0.034
Noninvasive ventilation	3.78	1.06-13.47	0.041
Polymicrobial infection	4.16	1.18-14.69	0.027
Lactic acid >2 mmol/L	0.84	0.25-2.87	0.785
Metastatic stage	1.9	0.54-6.72	0.318

Infection severity outcome

To further explore heterogeneity in clinical severity, additional stratified analyses were performed (Table 6). Culture-positive infection episodes were associated with an ICU admission rate of 26.8% (94/351), compared with 19.9% (45/226) among culture-negative episodes; this difference was not statistically significant (p = 0.0765). Severe neutropenia (ANC <500/μL) was present in 28 episodes (4.9%); among these, the ICU admission rate was 32.1% (9/28), compared with 23.7% (130/549) in episodes without severe neutropenia. This difference likewise did not reach statistical significance (p = 0.4288), likely reflecting limited statistical power due to the small number of neutropenic events.

**Table 6:** Stratified analysis by culture status and neutropenia severity Statistical tests: ^*^ chi-square test; ^†^ Fisher’s exact test Findings are exploratory due to the limited sample size in subgroups.

Variable	Total (n = 577)	ICU admission, n (%)	No ICU, n (%)	p-Value
Culture status				0.0765^*^
Culture-positive	351	94 (26.8)	257 (73.2)	
Culture-negative	226	45 (19.9)	181 (80.1)	
Severe neutropenia (≤500 cells/mm³)				0.4288^†^
Yes	28	9 (32.1)	19 (67.9)	
No	549	130 (23.7)	419 (76.3)	

Subgroup analyses

No significant difference in 28-day mortality was observed between patients with hematologic malignancies (16/117, 13.7%) and solid tumors (88/460, 19.1%; p = 0.216). The small number of hematologic patients (n = 30, 15.1%) limits statistical power; these findings are exploratory. Vasopressor use was significantly higher among solid tumor patients (25/460, 5.4% vs. 0/117, 0%; p = 0.004). Combination anticancer therapy showed a trend toward higher mortality (33/134, 24.6%) compared with single-modality therapy (64/383, 15.7%; p = 0.067). Resistant infections (49/577, 8.5%) were associated with increased ICU admission (17/49, 34.7% vs. 120/528, 22.7%; p = 0.07) but similar 28-day mortality (8/49, 16.3% vs. 63/528, 11.9%; p = 0.38) compared with susceptible infections.

Subgroup analysis of 28-day mortality using Kaplan-Meier survival analysis showed that terminal illness (DNR status) was associated with significantly lower 28-day survival (log-rank p < 0.001). Severe neutropenia showed a trend toward worse survival (p = 0.059), while metastatic status (p = 0.729) and known infection site (p = 0.575) were not significantly associated with 28-day mortality.

Antibiotic usage

Among 533 episodes (92.4%) with available data, the most frequently prescribed antibiotics were piperacillin/tazobactam (229, 43.0%), ciprofloxacin (152, 28.5%), and meropenem (146, 27.4%) (Table 7). Antifungal agents were used in 34-41 episodes (6-8%). Data on antifungal prophylaxis or G-CSF were not available. In exploratory univariate analyses, the use of piperacillin/tazobactam was associated with higher 28-day mortality (18.2% vs. 8.9%; p = 0.002), as was carbapenem use (19.4% vs. 9.9%; p = 0.004). A higher number of antibiotics was also associated with increased ICU admission (31.9% for ≥3 antibiotics vs. 17.2% for 0-1; p = 0.005).

**Table 7:** Antibiotic usage (episode level, N = 533) Data are presented for 533 episodes (92.4%) with complete antibiotic documentation. Patients may have received multiple antibiotics; percentages sum to >100%. Data on antifungal prophylaxis or G-CSF use were not available. G-CSF, granulocyte colony-stimulating factor

Antibiotic class	Antibiotic	Frequency (n)	Percentage (%)
Penicillins	Piperacillin/tazobactam	229	43
	Amoxicillin/clavulanate	100	18.8
	Ampicillin	7	1.3
	Amoxicillin	6	1.1
Carbapenems	Meropenem	146	27.4
	Imipenem	32	6
Fluoroquinolones	Ciprofloxacin	152	28.5
	Levofloxacin	103	19.3
	Moxifloxacin	38	7.1
Cephalosporins	Ceftriaxone	83	15.6
	Cefazolin	22	4.1
	Ceftazidime	17	3.2
	Cefuroxime	16	3
	Cefepime	13	2.4
	Cefotaxime	11	2.1
Other antibiotics	Metronidazole	81	15.2
	Linezolid	66	12.4
	Vancomycin	29	5.4
	Tigecycline	16	3
	Amikacin	16	3
	Colistin	10	1.9
	Clindamycin	10	1.9
	Cotrimoxazole	6	1.1
Antifungals	Anidulafungin	34	6.4
	Caspofungin	21	3.9
	Miconazole	11	2.1
	Fluconazole	8	1.5

Hospital length of stay (LOS)

Median hospital LOS was eight days (IQR 5-8) among 556 episodes (96.4%) with available data. Survivors had a median LOS of eight days (IQR 4-8), while non-survivors also had a median LOS of eight days (IQR 8-8). The narrow IQR among non-survivors suggests possible truncation bias.

Recurrent infections

Recurrent infections occurred in 106 patients (55%), with a median of two episodes per patient (range 1-28). The Andersen-Gill extension of the Cox model accounted for within-patient clustering, and sensitivity analyses using frailty terms did not significantly improve model fit (p > 0.05).

## Discussion

In this retrospective study of cancer patients from Najran, Saudi Arabia, we characterized infection patterns and identified key predictors of 28-day mortality. Gram-negative organisms and polymicrobial infections were major drivers of mortality, with independent predictors including ICU admission, neutropenia, noninvasive ventilation, and polymicrobial infection. To our knowledge, this is the first detailed description of infection-related mortality in southern Saudi Arabia, supporting the implementation of localized antimicrobial stewardship and severity-based triage strategies.

The MDRO rate in our cohort was 8.5%, substantially lower than the 40-60% prevalence reported in localized oncology cohorts from Riyadh, Jeddah, and other GCC settings [16-18], as well as the 37% MDR rate documented in a Greek cancer cohort [19]. While this discrepancy might suggest lower cumulative antibiotic pressure in the Southern Province compared with high-volume tertiary centers, it could also be influenced by differences in laboratory practices, culture techniques, or underreporting of resistant isolates. Possible explanations include incomplete microbiological workup, selective culturing of low-risk patients, or limited use of molecular resistance detection methods. Therefore, the low rate should be interpreted with caution; it may not fully represent the true burden of antimicrobial resistance in this population. Without prospective validation and standardized surveillance, we cannot exclude the possibility that the observed rate is, at least in part, an artifact of diagnostic or documentation gaps.

Gram-negative bacteria predominated in our cohort, with *Klebsiella *species (12.0%) and *E. coli *(11.1%) most frequently isolated. This aligns with recent trends in Saudi Arabia and internationally. Alharbi et al. reported that Gram-negative bacteria comprised 76% of infections at Princess Noorah Oncology Center, with *Klebsiella pneumoniae* as the most common isolate (35.3%) [20]. Similarly, Ntim et al. identified *E. coli*, *K. pneumoniae*, *Pseudomonas aeruginosa*, and *Acinetobacter baumannii *as major pathogens in cancer patients worldwide [21], while Kayaaslan et al. reported similar findings [22]. Among the 42 *E. coli *and *Klebsiella *isolates tested for ESBL at the clinician’s discretion (representing only 32% of such isolates), 12 (28.6%) were ESBL-producing. Because testing was not systematic, this proportion may not reflect the true prevalence; ongoing surveillance with standardized protocols is needed. The occurrence and distribution of bacterial infections are influenced by factors including the severity and length of neutropenia, the aggressiveness of antineoplastic treatment, the use of antimicrobial prophylaxis, the presence of central venous catheters, environmental conditions, and the length of hospital stay [23].

In our study, the antibiotics most commonly prescribed were piperacillin/tazobactam (43.0%), ciprofloxacin (28.5%), and meropenem (27.4%). The high use of broad-spectrum agents, particularly carbapenems, likely reflects the severity of illness and the high proportion of culture-negative episodes (39.2%) where empirical coverage is necessary. However, given the low observed MDRO rate (8.5%), this level of broad-spectrum antibiotic use may be excessive and could contribute to future resistance selection. Published stewardship data suggest that reducing carbapenem and glycopeptide use can be achieved without increasing other broad-spectrum agents and may be associated with trends toward lower rates of ESBL-producing Enterobacterales and carbapenem-resistant *P. aeruginosa*, as well as reduced vancomycin-related nephrotoxicity and VRE colonization [24]. Antifungal medications were utilized in only 6-8% of cases, indicating a relatively low suspicion of invasive fungal infections within this group. Implementing prospective antimicrobial stewardship programs could aid in justifying empirical treatment, particularly for carbapenems, while maintaining the currently favorable resistance patterns.

Polymicrobial infections occurred in 7.3% of episodes and independently predicted mortality (adjusted HR 4.16). The high HR likely reflects the challenge of selecting empirical regimens that adequately cover all pathogens in a polymicrobial mix, potentially delaying appropriate therapy. This is consistent with evidence that multiple pathogens confer worse outcomes due to synergistic virulence and treatment challenges [13]. Marín et al. reported comparable findings, documenting inadequate empirical antibiotic therapy in 23% of cancer patients with bacteremia, with a markedly higher proportion (69%) among those infected with MDR strains [25]. In Perdikouri et al.’s study, the vast majority (87%) did not receive effective empirical antibiotics, despite 69% receiving some form of empirical therapy [19].

A major clinical concern was the absence of a clinically localized infection site in 76.3% of episodes. Among these 440 episodes, 254 (57.7%) had positive blood cultures, and imaging was performed in 312 (70.9%), yet a focal source was identified in only 98 (31.4%) of those imaged. Among localized cases, urosepsis (10.9%) and pneumonia (5.7%) predominated, which aligns with the known distribution of febrile neutropenic episodes (pneumonia, gastrointestinal, skin/soft tissue, and oral cavity) [26,27].

This finding must be interpreted in the context of our cohort: the majority had advanced/metastatic disease (66.8%), and a substantial proportion of episodes were hospital-acquired (47.5%) or occurred in neutropenic patients (11.4%). Contributing factors include neutropenia-related blunting of inflammatory responses, immunosuppression masking fever and pain, tumor-related obstruction creating cryptic foci, and prior antibiotic use impairing pathogen isolation [23,28].

Our findings align with other oncology cohorts: a Swiss study of hospital-acquired bloodstream infections reported a nearly identical 75% “unknown/other source” rate among hematology patients, and a Spanish cohort found approximately 57% of infections classified as unknown source [29,30]. Some of this proportion may reflect a diagnostic gap, particularly early antibiotic initiation before comprehensive localization, but advanced disease itself likely contributes [28,31]. With retrospective data, we cannot disentangle the relative contributions of diagnostic limitation versus disease-related masking. Nevertheless, improving diagnostic ascertainment through structured infection workup bundles remains actionable, as earlier pathogen identification directly informs empirical therapy selection and de-escalation, especially given the high HR for polymicrobial infections and the documented link between inadequate empirical therapy and mortality in GCC data [32,33].

The 28-day mortality rate was 12.3% per episode, lower than rates reported in other oncological populations (e.g., 32% in Perdikouri et al. [19] and 32% in Marín et al. [25]), likely reflecting our low MDRO prevalence and a high proportion of DNR orders (67.6% among non-survivors), indicating that many deaths occurred in the context of advanced disease with comfort-focused care [34]. The role of infection as a direct contributor to mortality in this population remains complex. Cause-of-death analysis showed that a substantial proportion of deaths were attributed to malignancy progression, with additional cases categorized as organ failure or treatment-related complications. This overlap underscores the difficulty of distinguishing infection-attributable mortality from overall disease trajectory in oncology populations. Therefore, the associations identified in this study should be interpreted within the context of all-cause mortality rather than as direct causal effects of infection.

ICU admission was the strongest mortality predictor (adjusted HR 8.56), consistent with literature showing that severe infections requiring critical care portend poor outcomes in oncology patients [10,35,36]. Noninvasive ventilation also independently predicted mortality, highlighting respiratory failure as a serious prognostic marker even without intubation.

Traditional risk factors such as older age and comorbidity burden did not significantly impact 28-day mortality, despite 66.8% of patients having metastatic disease. This may reflect limited statistical power or that the 28-day window is too short for chronic disease burden to manifest fully, with acute physiological derangement dominating short-term outcomes. The loss of significance for metastatic stage and lactic acid after adjustment suggests their effects are mediated through organ failure and ICU admission [10,34]. Clinicians should therefore maintain high vigilance when infection is suspected, regardless of baseline prognosis. Notably, our previous report on ICU outcomes in oncology patients identified different predictors (older age, advanced stage, respiratory infections, and higher SOFA scores) [10], likely due to differences in the study population (ICU-only vs. all hospital episodes) and the greater weight of acute organ dysfunction in the ICU setting.

Regarding cancer type, we observed no significant mortality difference between solid and hematologic tumors (p = 0.216), although the small number of hematologic cases (15.1%) limited statistical power. This may also reflect improvements in supportive care (prophylactic antimicrobials and growth factors) that have narrowed the outcome gap between these populations.

Limitations

This single-center, retrospective cohort study has a number of limitations. The use of a retrospective design and dependence on electronic medical records may have introduced bias or incomplete data. The patient sample size (N = 199) restricts statistical power for subgroup analyses, especially concerning hematologic malignancies and uncommon infection types. Furthermore, detailed data on specific anticancer treatment regimens (e.g., type, intensity, and timing of chemotherapy) and individual cancer subtypes were not sufficiently granular to allow meaningful adjustment; these factors could influence infection risk and outcomes but could not be fully accounted for due to limited sample size and collinearity with other variables. Collinearity was assessed using Spearman correlation, and variables with r > 0.7 were excluded from multivariable models.

The significant percentage of clinically unspecified infection sites might lead to an underestimation of actual pathogen prevalence; however, the blood culture positivity rate (57.7%) and imaging yield (70.9% for unlocalized episodes) provide some context. ESBL testing was conducted on a convenience sample of *E. coli* and *Klebsiella *isolates (32% of all such isolates), meaning that the 28.6% ESBL rate should be viewed as exploratory rather than conclusive. Information on antifungal prophylaxis and G-CSF usage was not available.

DNR status was documented, but its timing relative to infection onset and its influence on treatment intensity could not be fully disentangled; similarly, objective measures of disease severity (e.g., SOFA or APACHE scores) were not consistently recorded, limiting adjustment for baseline physiological derangement. Most critically, it was not possible to reliably differentiate infection-related mortality from overall mortality, with over half of deaths (51.0%) attributed to cancer progression, thus diminishing any direct correlation between antimicrobial stewardship and a decrease in overall mortality.

Future prospective multicenter studies that utilize standardized definitions, thorough microbiological testing, and time-dependent analyses are essential to further clarify the relationship between infection and malignancy in this group.

## Conclusions

In this 10-year cohort of cancer patients with infection episodes in Najran, markers of acute clinical deterioration, such as ICU admission (as a severity marker), noninvasive ventilation, neutropenia, and polymicrobial infection, were independently associated with 28-day all-cause mortality. While Gram-negative pathogens predominate, the region maintains a low multidrug resistance rate (8.5%). However, more than half of all deaths (51.0%) were attributed to cancer progression rather than infection, and a substantial proportion of organ failure-related deaths could not be definitively ascribed to infection; therefore, the potential impact of antimicrobial stewardship on overall mortality is likely limited to the subset of patients with potentially reversible infection-related organ failure. Given the retrospective, single-center nature of this study, the findings should be interpreted cautiously and validated in prospective multicenter research. Preserving the favorable low resistance profile remains a worthwhile goal. In the resource-constrained Najran setting, pragmatic, low-cost interventions, such as ensuring timely blood cultures, early imaging when clinically indicated, and developing local antibiograms, may improve diagnostic accuracy and antimicrobial use without requiring major infrastructure expansion. Local antimicrobial guidelines should prioritize preservation of last-line agents, leveraging the current low-resistance window. These recommendations are exploratory and require prospective validation.
